# Improved treatment and prognosis after acute myocardial infarction in Estonia: cross-sectional study from a high risk country

**DOI:** 10.1186/s12872-015-0129-7

**Published:** 2015-10-26

**Authors:** Aet Saar, Toomas Marandi, Tiia Ainla, Krista Fischer, Mai Blöndal, Jaan Eha

**Affiliations:** Department of Cardiology, University of Tartu, Tartu, Estonia; Centre of Cardiology, North Estonia Medical Centre, Tallinn, Estonia; Estonian Genome Centre, University of Tartu, Tartu, Estonia; Heart Clinic, Tartu University Hospital, Tartu, Estonia

**Keywords:** Acute myocardial infarction, Treatment, Mortality rates, Estonia

## Abstract

**Background:**

The aim of the study was to explore trends in short- and long-term mortality after hospitalization for acute myocardial infarction (AMI) over the period 2001─2011 in Estonian secondary and tertiary care hospitals while adjusting for changes in baseline characteristics.

**Methods:**

In this nationwide cross-sectional study random samples of patients hospitalized due to AMI in years 2001, 2007 and 2011 were identified and followed for 1 year. Trends in 30-day and 1-year all-cause mortality were analysed using Cox proportional hazards regression model.

**Results:**

The final analysis included 423, 687 and 665 patients in years 2001, 2007 and 2011 respectively. During the study period, the prevalence of most comorbidities remained unchanged while the in-hospital and outpatient treatment improved significantly. For example, the proportion of tertiary care hospital AMI patients who underwent revascularization was almost three times higher in 2011 compared to 2001. The proportion of secondary care patients who were referred to a tertiary care centre for more advanced care increased from 5.8 to 40.1 % (*p* for trend <0.001). Meanwhile, the 1-year mortality rates decreased from 29.5 to 20.2 % (adjusted *p* = 0.004) in the tertiary and from 32.4 to 23.1 % (adjusted *p* = 0.006) in the secondary care. The decrease in the 30-day mortality rates was statistically significant only in the secondary care hospitals.

**Conclusions:**

The use of evidence-based treatments in Estonian AMI patients improved between 2001 and 2011. At the same time, we observed a significant reduction in the long-term mortality rates, both for patients primarily hospitalized into secondary as well as into tertiary care hospitals.

## Background

Coronary artery disease (CAD) is currently the number one cause of death in Europe [1]. Even though the fatality rates for acute myocardial infarction (AMI) have markedly decreased during the last few decades, Estonian death rates from CAD are still among the highest in Europe [2]. Modelling studies from different European countries have attributed declining trends in cardiovascular mortality to improved treatment and changes in cardiovascular risk factors [3, 4].

Important components of AMI treatment are early diagnosis, timely reperfusion and use of evidence-based medications [5, 6]. Earlier studies [7–9] from Estonia show improvement in AMI treatment, emphasizing better access to invasive diagnostics and treatment and wider use of evidence-based medications over time. However, no significant improvement in short- and long-term mortality in Estonian AMI population was seen [8, 9]. A recent overview about quality of care and mortality following AMI from Central and Eastern European countries describes lack of comparable data and wide variation in acute cardiac care, in both between and within European countries [10].

The aim of the present study is to explore trends in short- and long-term mortality rates after hospitalization for AMI over the period 2001─2011 in Estonian secondary and tertiary care hospitals while considering changes in baseline characteristics and treatment.

## Methods

**Fig. 1:**
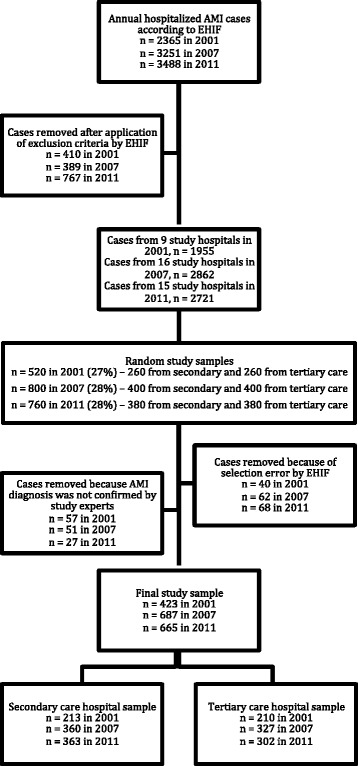
Formation of the study sample. AMI – acute myocardial infarction, EHIF – Estonian Health Insurance Fund

We conducted a nationwide cross-sectional study based on hospital records. The formation of the study sample is described in Fig. 1.

The list of all the AMI cases for each year was obtained from the Estonian Health Insurance Fund (EHIF) database. During the time period studied, approximately 95 % of the Estonian population was covered with the health insurance. The validity of AMI diagnoses in EHIF database has been established previously [11]. According to the EHIF database, the total number of AMI cases hospitalized (main diagnosis code I21─I22 according to the International Statistical Classification of Diseases [ICD] and Related Health Problems 10th revision) was as 2365 in 2001, 3251 in 2007 and 3488 in 2011. All Estonian hospitals are using ICD codes I21─I22 (acute and subsequent myocardial infarction) with extension to diagnose AMI. As we intended to evaluate treatment of AMI in the hospital where the patient was primarily hospitalized, the following exclusion criteria were applied: (1) patients who were not primarily hospitalized into one of the study hospitals; (2) patients who were re-admitted with AMI diagnosis within 28 days after the first admission (only the second admission was excluded); (3) patients whose length of hospital stay was less than 3 days if they were discharged alive and were not transferred, which made the diagnosis of AMI very unlikely considering the local clinical practice.

From the remaining cases a study sample was formed by the use of random selection. The sampling was performed in clusters in order to get cross-sectional overview from all cases admitted into different types of hospitals. To ensure data comparability across years, the formation of the study sample was similar in all years studied.

In order to have a representative sample of all Estonian AMI patients, we included hospitals that treat the major proportion of annual AMI cases. In 2001, there were two tertiary and seven secondary care hospitals responsible for the treatment of most AMI cases in Estonia. The tertiary care hospitals had percutaneous coronary intervention (PCI) availability during working hours, while the secondary care hospitals did not have PCI availability. In 2007, the study included sixteen hospitals, two of tertiary and fourteen of secondary care. By the year 2007, both tertiary care hospitals had 24/7 PCI availability and one secondary care hospital had PCI available during working hours. In 2011, the study included thirteen secondary and two tertiary care hospitals. The tertiary care hospitals and one secondary care hospital had 24/7 PCI availability and two secondary care hospitals had PCI availability once a week. Thus, ten out of thirteen secondary care hospitals did not have PCI availability.

In Estonia there are two tertiary care hospitals, which did not change during the study period. The number of secondary care hospitals varied in the years studied due to the restructuring of the hospital network of Estonia. Also, it should be noted, that the recommendations of the Estonian Society of Cardiology to admit patients with ST-segment elevation myocardial infarction (STEMI) for primary PCI to two tertiary care hospitals remained consistent during the study period.

The criteria applied for AMI diagnosis on 2001 and 2007 study populations were based on the consensus document published by the European Society of Cardiology in 2000 [12]. For 2011 cohort, the criteria were based on the redefinition of myocardial infarction published in 2007 [13]. In the data abstraction process, the medical records from study hospitals were obtained and data were collected retrospectively by experts according to the acute coronary syndromes data standards that were later presented in the CARDS Project [14]. The experts were certified cardiologists or cardiologists in training and all had received additional training on the data collection for this study. Every AMI case was reviewed by one expert, which was followed by random re-abstraction by another expert for data quality monitoring purposes. If discrepancies were determined, the experts were additionally trained. Data on mortality were obtained from the Estonian Population Registry. As the aim of the study was to evaluate the quality of care of the first hospital where the patient was admitted, data collection stopped after the patient was referred from a secondary care to a tertiary care hospital. Data on discharge medications were available only for those secondary care patients who were not referred to a tertiary care hospital.

The study was approved by the Research Ethics Committee of the University of Tartu.

### Statistical analysis

For all patient characteristics and outcome variables of interest, comparisons between three years (2001, 2007, 2011) and two types of hospital (tertiary vs secondary care) of primary hospitalization were made.

Categorical variables were summarized by percentages and continuous variables by means and standard deviations. Differences in continuous variables were examined by classical linear regression and differences in binary variables by logistic regression. Categorical variables with more than two categories were analysed using the Chi-Square test.

As main outcome variables in this study, 30-day and 1-year all-cause mortality was analysed. In addition to crude mortality rates, baseline adjusted (age, sex, AMI subtype, diabetes, hypertension, previous heart failure, previous myocardial infarction) rates were compared using the Cox proportional hazards regression model. Patients initially hospitalized into a secondary care hospital but transferred and treated in tertiary care hospital, were included in the mortality analysis as secondary care patients. Two sided P values <0.05 were considered statistically significant.

For all statistical analyses, R software (ver. 3.1.1) was used [15].

## Results

Final study sample included 423, 687 and 665 cases from years 2001, 2007 and 2011 respectively.

### Baseline characteristics

Baseline characteristics are presented in Tables 1 and 2. Although the mean age of the study sample increased in both hospital types during the period, there were no significant changes in the frequency of most comorbidities. The results show increased proportion of patients with non-ST-segment-elevation acute myocardial infarction (NSTEMI) compared to that of patients with STEMI in both hospital types over time.

**Table 1:** Baseline characteristics of acute myocardial infarction patients hospitalized primarily into tertiary care hospitals

	Year 2001	Year 2007	Year 2011	*P* value for trend
	*n* = 210	*n* = 327	*n* = 302	
Hospital days, mean, (SD)	11.4 (9.1)	10.0 (8.4)	9.2 (6.5)	0.002
Mean age (SD), years	68.3 (12.7)	69.7 (12.0)	71.0 (12.0)	0.015
≥75 years, %	31.0	37.0	41.4	0.017
Men, %	66.7	58.1	62.3	0.3
STEMI, %	61.9	49.5	53.0	0.043
Diabetes, %	19.0	27.2	26.2	0.065
Arterial hypertension, %	70.0	70.0	75.2	0.206
Previous MI, %	29.5	29.4	29.1	0.925
Previous heart failure, %	27.1	28.1	25.2	0.626
Time to presentation, %				
≤ 3 h	47.6	41.9	44.7	0.723
3–12 h	23.8	24.8	23.2	
> 12 h	28.6	33.3	32.1	

*MI* myocardial infarction, *STEMI* ST-segment elevation myocardial infarction, *SD* standard deviation

**Table 2:** Baseline characteristics of AMI patients hospitalized primarily into secondary care hospitals

	Year 2001	Year 2007	Year 2011	*P* value for trend
	*n* = 213	*n* = 360	*n* = 363	
Hospital days, mean, (SD)	11.4 (6.8)	9.4 (7.6)	6.5 (6.3)	<0.001
Mean age (SD), years	68.4 (12.4)	71.8 (11.4)	72.8 (12.2)	<0.001
≥75 years, %	34.3	45.3	47.4	0.002
Men, %	52.1	51.9	48.5	0.4
STEMI, %	59.6	51.4	44.4	<0.001
Diabetes, %	16.4	31.1	21.5	0.225
Arterial hypertension, %	57.3	75.8	74.7	<0.001
Previous MI, %	23.9	27.2	30.9	0.073
Previous heart failure, %	26.8	31.7	32.2	0.176
Time to presentation, %				
≤ 3 h	31.0	30.6	30.0	0.993
3–12 h	25.8	25.0	26.4	
> 12 h	43.2	44.4	43.5	

*MI* myocardial infarction, *STEMI* ST- segment elevation myocardial infarction, *SD* standard deviation

### Treatment

Guideline-recommended treatments were more likely to be used for patients hospitalized in 2011 than in the earlier years in both hospital types (Tables 3 and 4). Cardiac catheterization and percutaneous revascularization became a dominant strategy in the tertiary care setting. The reperfusion rates for STEMI increased from 42.3 to 63.1 % (*p* < 0.001) in the tertiary care hospitals, while there was no statistically significant change in the secondary care hospitals. Meanwhile, there was an important increase in the proportion of patients who were referred from a secondary to a tertiary care hospital for further diagnostics and treatment (from 5.8 to 40.1 %, *p* < 0.001). The prescription rates of cardiovascular medications recommended by guidelines at discharge increased in all five drug groups in both hospital types (Table 5).

**Table 3:** In-hospital management in tertiary care hospitals

	Year 2001	Year 2007	Year 2011	*P* value for trend
	*n* = 210	*n* = 327	*n* = 302	
	%	%	%	
Medications				
Aspirin	87.1	94.2	94.4	0.003
P2Y_12_-receptor inhibitors	17.1	61.5	70.5	<0.001
Anticoagulants	89.0	93.0	92.7	0.133
Glycoprotein IIb/IIIa inh.	12.4	38.8	29.1	<0.001
Betablockers	79.5	82.6	82.1	0.452
Nitrates	92.4	78.9	76.2	<0.001
ACEi/ARB	70.5	74.9	81.1	0.006
Statins	26.7	67.9	77.2	<0.001
Cardiac catheterization	35.7	78.6	80.8	<0.001
Revascularization	27.6	64.2	73.5	<0.001
PCI	22.4	61.5	67.9	<0.001
CABG	5.2	3.7	6.0	0.722
Echocardiography	81.9	85.3	88.4	0.044
Treatment for STEMI	*n* = 130	*n* = 162	*n* = 160	
Reperfusion for STEMI	42.3	64.2	63.1	<0.001
Thrombolysis	35.4	7.4	0.6	<0.001
Primary PCI	6.9	56.8	62.5	<0.001
Treatment for NSTEMI	*n* = 80	*n* = 165	*n* = 142	
PCI	18.8	47.9	53.5	<0.001
CABG	7.5	3.6	9.2	0.56

Anticoagulants – low molecular weight heparins/unfractionated heparin/ fondaparinux, *ACEi* angiotensin-converting enzyme inhibitors, *ARB* angiotensin II receptor blockers, P2Y_12_-receptor inhibitors – ticlopidine/clopidogrel/ticagrelol, *CABG* coronary artery bypass grafting, *PCI* percutaneous coronary intervention, *STEMI* ST- segment elevation myocardial infarction, *NSTEMI* non-ST-segment elevation myocardial infarction

**Table 4:** In-hospital management in secondary care hospitals

	Year 2001	Year 2007	Year 2011	*P* value for trend
	*n* = 213	*n* = 360	*n* = 363	
	%	%	%	
Medications				
Aspirin	88.3	86.4	85.7	0.383
P2Y_12_-receptor inhibitors	0	10.6	26.4	<0.001
Anticoagulants	85.4	92.8	95.0	<0.001
Glycoprotein IIb/IIIa inh.	0.5	3.1	5.2	0.003
Betablockers	76.1	77.8	73.0	0.384
Nitrates	96.7	85.6	78.8	<0.001
ACEi/ARB	37.1	62.2	55.9	<0.001
Statins	5.6	30.8	49.0	<0.001
Cardiac catheterization	0	6.7	18.5	<0.001
Revascularization	0	4.2	14.3	<0.001
PCI	0	4.2	14.3	<0.001
CABG	0	0	0	-
Echocardiography	52.1	51.9	50.7	0.735
Referred to a tertiary care hospital	5.8	24.8	40.1	<0.001
Treatment for STEMI	*n* = 127	*n* = 185	*n* = 161	
Reperfusion for STEMI	44.1	34.1	37.9	0.251
Thrombolysis	44.1	34.1	29.2	0.008
Primary PCI	0	0	8.7	-
Treatment for NSTEMI	*n* = 86	*n* = 175	*n* = 202	
PCI	0	4.6	10.4	0.002
CABG	0	0	0	-
Referred to a tertiary care hospital	5.8	10.3	28.2	<0.001

Anticoagulants – low molecular weight heparins/unfractionated heparin, fondaparinux, *ACEi* angiotensin-converting enzyme inhibitors, *ARB* angiotensin II receptor blockers, P2Y_12_-receptor inhibitors – ticlopidine/clopidogrel/ticagrelol, *CABG* coronary artery bypass grafting, *PCI* percutaneous coronary intervention, *STEMI* ST-segment elevation myocardial infarction, *NSTEMI* non-ST-segment elevation myocardial infarction

**Table 5:** Medications prescribed for outpatient use in tertiary and secondary care hospitals

	Year 2001	Year 2007	Year 2011	*P* value for trend
	%	%	%	
Tertiary care hospitals	*n* = 181	*n* = 290	*n* = 261	
Aspirin	85.1	93.1	95.4	<0.001
P2Y_12_-receptor inhibitors	18.2	64.8	72.8	<0.001
Betablockers	71.3	80.0	85.4	<0.001
ACEi/ARB	66.3	77.2	84.7	<0.001
Statins	31.5	73.4	80.8	<0.001
Nitrates	61.9	22.1	15.8	<0.001
Secondary care hospitals	*n* = 163	*n* = 224	*n* = 184	
Aspirin	79.8	82.6	91.3	0.004
P2Y_12_-receptor inhibitors	0.6	10.7	32.8	<0.001
Betablockers	68.7	80.9	82.8	0.001
ACEi/ARB	37.4	68.9	68.3	<0.001
Statins	14.7	37.3	65.6	<0.001
Nitrates	85.3	58.2	41.4	<0.001

*ACEi* angiotensin-converting enzyme inhibitors, *ARB* angiotensin II receptor blockers, P2Y_12_-receptor inhibitors – ticlopidine/clopidogrel/ticagrelol

### Mortality

There was a statistically significant decrease from 20.2 to 12.4 % (adjusted *p* = 0.003) in 30-day mortality rate in the secondary care setting during the period studied (Table 6). 30-day mortality reduction was not statistically significant in the tertiary care hospitals. Results from long-term mortality analysis show decrease from 29.5 to 20.2 % (adjusted *p* = 0.004) in the tertiary care and from 32.4 to 23.1 % (adjusted *p* = 0.006) in the secondary care hospitals in 1-year mortality rates.

**Table 6:** Mortality of acute myocardial infarction patients primarily hospitalized into tertiary and secondary care hospitals

Mortality	2001	2007	2011	*P* value for trend, unadjusted	HR (95 % CI) change per year, unadjusted	*P* value for trend, adjusted^a^	HR (95 % CI) change per year, adjusted^a^
	%	%	%				
30-day							
Tertiary care hospitals	17.6	13.1	13.2	0.181	0.97 (0.926–1.015)	0.061	0.96 (0.913–1.002)
Secondary care hospitals	20.2	22.5	12.4	0.022	0.96 (0.920–0.994)	0.003	0.94 (0.904–0.980)
1-year							
Tertiary care hospitals	29.5	24.5	20.2	0.026	0.96 (0.928–0.995)	0.004	0.95 (0.917–0.984)
Secondary care hospitals	32.4	35.0	23.1	0.026	0.97 (0.938–0.996)	0.006	0.95 (0.918–0.977)

^a^adjusted for age, sex, AMI subtype (STEMI vs NSTEMI), previous myocardial infarction, previous heart failure, diabetes, hypertension

*STEMI* ST-segment elevation myocardial infarction, *NSTEMI* Non-ST-segment elevation myocardial infarction, *HR* hazard ratio, *CI* confidence interval

**Fig. 2:**
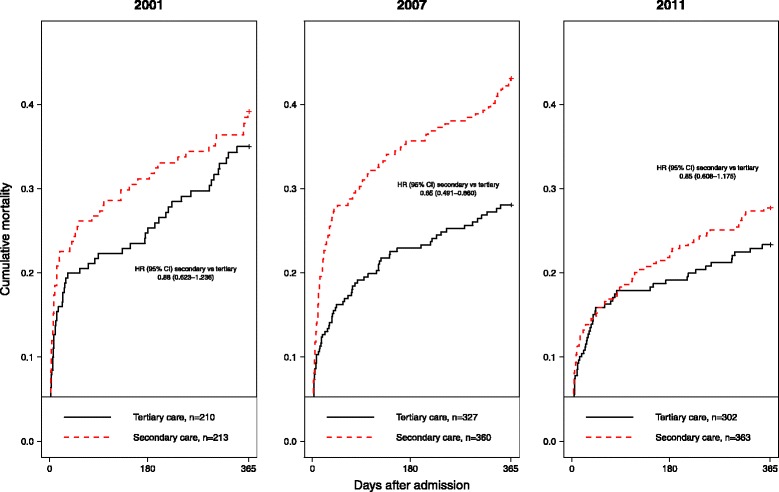
Cumulative mortality hazards of patients hospitalized primarily into secondary and tertiary care hospitals. HR – hazard ratio, CI – confidence interval

From the results of mortality analysis comparing different years and hospital types we found marked decline in mortality rates in both types of hospitals, which took place first in the tertiary and then in the secondary care. Mortality rates were similarly high in 2001. 30-day and 1-year mortality had decreased by year 2007 only in the tertiary care. By 2011, mortality rates had declined in both hospital types; mortality gap between the secondary and the tertiary care had disappeared (Fig. 2).

## Discussion

In this countrywide analysis covering period 2001─2011, we demonstrated a decrease in short- and long-term mortality of AMI patients. The mortality reduction is consistent with reports from other countries and is generally attributed to many factors, including improved risk factor management, more frequent use of pharmacological agents and more widespread availability of revascularization methods, especially primary PCI [3, 4, 16, 17]. Also, developments in efficacy and safety of coronary artery stents may have improved the outcome [18].

The prevalence of STEMI has decreased in both hospital types, which is counter-balanced by higher proportion of NSTEMI. Improved coronary risk factor management and treatment after first coronary event may have contributed to the observed trend [19]. Another plausible explanation is the rising mean age, which is consistent with earlier studies describing higher prevalence of NSTEMI among the elderly [20]. Third and probably the most important explanation for the growing ratio of NSTEMI to STEMI is the more widespread use of high-sensitivity troponin assays, which has resulted in more sensitive diagnostics [21].

During last decades, led by the Estonian Society of Cardiology, much effort has been offered to improve diagnostics and treatment of AMI. Quality improvement measures have targeted different aspects of the AMI management, including prehospital triage and establishing STEMI network, therapies during hospitalization, at discharge and outpatient care. For example, local STEMI guideline was published [22], European AMI definitions and guidelines were translated into Estonian and several educational events throughout Estonia were organized. At the same time, access to cardiac catheterization facilities has improved.

Reperfusion rates for STEMI are used as performance measures of AMI treatment. Findings indicate that reperfusion rates in the tertiary care hospitals are now comparable with respective rates form North, West, and Central Europe [23, 24]. Results are different for the secondary care – only approximately 40 % of STEMI patients are being offered reperfusion, with no increase during the period studied. However, low reperfusion rates should be interpreted with caution – in 2011 more than 40 % of secondary care patients were referred to a tertiary care centre for further management. We can hypothesize that patients were transferred before receiving reperfusion. Nevertheless, such trend is alarming, because transfer increases the delays and timely PCI is impossible. Data from international EPICOR registry suggest that recommended times are often not met when AMI patients are transferred for primary PCI [25]. Primary PCI is recommended as first line therapy for STEMI but it should be emphasized that thrombolysis is also an appropriate and proven reperfusion strategy [26]. However, more frequent referral to the tertiary care hospitals is in agreement with guidelines that recommend an invasive management for STEMI or high-risk NSTEMI patients [7, 8]. Also, local quality improvement initiatives have stressed the importance of timely referral of STEMI patients without contraindications and most NSTEMI patients to tertiary care centres with catheterization laboratories.

In addition to the reperfusion therapy, the recommended concomitant pharmacological therapy and the discharge medications play a major role in determining prognosis. Lower prescription rates of secondary prevention drugs in the secondary care can be partly explained by differences in the baseline characteristics. Patients in the secondary care hospitals were older and it has been shown that elderly patients are less likely to receive medications recommended by guidelines [27]. Previously described lower adherence to guidelines in smaller non-academic hospitals, staffed less frequently with certified cardiologists, is another plausible explanation [28, 29]. However, utilization rates of recommended drugs in the tertiary care hospitals are similar with corresponding rates from the UK, Sweden, and the US [30]. Also, patient compliance with suggested medications plays an important role in determining the prognosis. Failure to adhere to suggested therapies leads to more frequent hospital readmissions and has a negative impact on mortality [31, 32]. Similar problems related to the compliance with suggested drugs after AMI have been previously described in Estonia [33].

Another noteworthy finding from the study is the big proportion of patients who present late after symptom onset. Longer ischaemic times are associated with more myocardial damage and have adverse impact on outcome [34, 35]. Approximately 43 % of patients who present later than 12 h after symptom onset explain why reperfusion rates have remained low in the secondary care hospitals. Unfortunately, presentation delays did not show decrease over time.

Treatment of AMI patients improved mainly in the tertiary care hospitals with the main changes occurring during the first part of the study period. Inconsistency in the speed of implementation of new treatment strategies was reflected as a marked mortality gap in 2007 between the secondary and the tertiary care hospitals. By the 2011, the differences in treatment persisted, but a significant proportion of patients were transferred for further management to the tertiary care hospitals. Consequently, the noticeable mortality gap between the secondary and the tertiary care hospitals was no longer present in 2011. A similar initial large variation in treatments between different hospitals and gradual lowering of short- and long-term mortality are previously described in Sweden for period 1996─2007 [36] and in the US between 1995 and 2006 [37].

The present study has several limitations. The first limitation is that it cannot prove clear causality of observed decrease in the mortality rates. Through adjusting for baseline characteristics, we can reduce the possibility that differences in the patient population accounted for the change, but causality between practice patterns and outcomes should not be inferred. Secondly, the present study describes three random patient samples from studied years, thus not describing the complete AMI population for the period. Consequently, the treatment regimens in the present study may not be exactly as of the whole AMI population. Thirdly, we did not collect information about contraindications to certain treatments; therefore, we were not able to evaluate how big proportion of eligible patients received recommended treatment. Fourthly, we did not collect data about drug compliance and utilization of other secondary prevention methods including smoking cessation etc. The importance of compliance with recommended antiplatelet therapy after coronary stent implementation is highlighted in recent study, which describes almost 5-fold increase in cardiac mortality rates for patients who discontinued clopidogrel within 3-months after the PCI procedure [38]. Thus, we were unable to account for the effect of these or any other unmeasured confounders, which might have influence to the long-term outcome.

## Conclusions

In this country-wide analysis covering period 2001─2011, we reported a decrease in 1-year mortality of AMI patients. During the study period, prevalence of most comorbidities remained unchanged while AMI management improved significantly. Guideline-recommended acute phase treatments were increasingly used in the tertiary care setting. Secondary care hospitals are still lagging behind, but substantial amount of patients are now referred to a tertiary care centre for more advanced care. In conclusion, we were able to demonstrate improved prognosis for Estonian AMI patients during the decade from 2001 to 2011. Furthermore, we determined that the prognosis does not depend on the hospital type where patient is hospitalized primarily – by the end of the study period, Estonian hospitals were functioning as an efficient network, delivering quite equal care to AMI patients as it was aimed by the Estonian Society of Cardiology.
